# Publisher Correction: Estimating and explaining the spread of COVID-19 at the county level in the USA

**DOI:** 10.1038/s42003-021-01679-0

**Published:** 2021-01-20

**Authors:** Anthony R. Ives, Claudio Bozzuto

**Affiliations:** 1 Department of Integrative Biology, University of Wisconsin-Madison, Madison, WI 53706 USA; 2 Wildlife Analysis GmbH, Oetlisbergstrasse 38, 8053 Zurich, Switzerland

**Keywords:** Infectious diseases, Ecological epidemiology, Evolutionary genetics

Correction to: *Communications Biology* 10.1038/s42003-020-01609-6, published online 5 January 2021.

In the original version of the published manuscript, there was an error in Eq. 2 in which the tau was misplaced and denoted as a multiplier of the distribution of the proportion of secondary infections caused by a primary infection that occurred τ days previously (1) rather than as a power to which r(t) is raised (2)

1. Original version $$R\left( t \right) = 1{\mathrm{/}}\mathop {\sum}\nolimits_\tau {{\mathrm{e}}^{ - r(t)}\tau p(\tau )}$$
2. Corrected version $$R\left( t \right) = 1/\mathop {\sum}\nolimits_\tau {{\mathrm{e}}^{ - r(t)\tau }p(\tau )}$$

