# Supplementary material for: Estimating and explaining the spread of COVID-19 at the county level in the USA
Source: Commun Biol. 2021 Jan 5;4:60. doi: 10.1038/s42003-020-01609-6 (PMC7785728; doi:10.1038/s42003-020-01609-6)
Supplement: Supplementary file 2 — Supplementary Information [file 42003_2020_1609_MOESM2_ESM.pdf]

Supplementary Information

Estimating and explaining the spread of COVID-19 at the county level in the USA

Anthony R. Ives[1 *], Claudio Bozzuto[2]

Affiliations:

[1] Department of Integrative Biology, University of Wisconsin-Madison, Madison, WI 53706, USA. arives@wisc.edu. ORCID 0000-0001-9375-9523.

[2] Wildlife Analysis GmbH, Oetlisbergstrasse 38, 8053 Zurich, Switzerland. bozzuto@wildlifeanalysis.ch. ORCID 0000-0003-0355-8379.

The Supplementary Information includes:

Supplementary Methods:

- Overview of Statistical Methods
- Simulation model
- Analysis of SARS-CoV-2 strains

Supplementary Figures 1–5

Supplementary Data 1 (metadata)

Supplementary Tables 1–4

Supplementary References

Supplementary Methods

Overview of Statistical Methods

The rate of spread of a disease in a population at the early phase of an epidemic, r_0, when the entire population is susceptible depends on the basic reproduction number, R_0, giving the number of secondary infections produced per infected individual, and the distribution of the time between primary and secondary infections. Thus, if the spread rate and distribution of infection times can be estimated, R_0 can then be calculated. Our strategy is to estimate r_0 as the most direct parameter associated with the dynamics of an epidemic, and then subsequently estimate R_0. The advantages of calculating r_0 include: (i) it captures all of the real-life complexities that affect R_0 by simply observing what happened in real life, and (ii) it uses data that are (tragically) becoming more prevalent. The challenges include (i) the changes in $r(t)$ that are to be expected (and hoped for) as people and governments respond to lessen the spread, and (ii) the statistical challenges and uncertainties of determining rates of disease spread when the numbers of deaths are still low.

We developed and tested statistical methods to overcome the two challenges of estimating R_0 from death data. Because the rate of spread of a disease may change rapidly in response to actions that are taken to reduce disease transmission, we used a time-varying autoregressive model that allows for the rate of spread to change through time, $r(t)$. Other models take a related approach[1,2]. The second challenge is that the counts of deaths at the beginning of an epidemic are low. To account for this, the time-series model includes increased uncertainty (measurement error) that depends on the time-varying estimate of the number of deaths. Standard (asymptotic) approaches often have poor statistical properties (type I errors, correctly calculated confidence intervals) when sample sizes are small[3]. Therefore, we use bootstrapping[4] in which simulation time series are reconstructed to share the same pattern as the observed time series; a large number of simulated time series are then fit using the same statistical model as used to fit the original data. This bootstrapping procedure thus gives estimates and confidence intervals for model fit to the real data. Note that our approach is frequentist, in comparison to the majority of models that use a Bayesian framework.

Our approach focuses on estimating the time-varying rate of spread, $r(t)$, of the number of deaths. Our rationale is that, for statistical fitting, it is better to keep the model as simple as

possible, rather than "building in" assumptions about the processes of infection, reporting, and death. Our simple phenomenological model uses the same data as more-complicated, process-based models, and therefore both approaches ultimately rely on the same information. The simpler approach, however, does not depend on assumptions about the infection processes. Instead, after estimating r_0, we computed R_0 as $1/\sum_\tau e^{-r(t)\tau}p(\tau)$, where τ is the number days after initial infection, and $p(\tau)$ is the proportion of secondary infections produced per infected individual at τ[5]. This expression assumes that deaths (removal of individuals from the population) occur after all secondary infections have occurred. We used the distribution of $p(\tau)$ that was estimated using contact tracing in Wuhan, China[6].

To validate the statistical method, we constructed a simulation model of the transmission process and spread of infections iterated on a daily time scale. Our simulations considered scenarios in which the transmission rate changed through time either in steps or gradually to capture the extremes of possible changes in real $R(t)$. We varied the initial R_0 and duration of simulations to produce epidemics that qualitatively match the county data we analyzed. Changes in our estimates of $r(t)$ tended to lag behind changes in the true (simulated) value of $r(t)$ (gray line and regions in Supplementary Fig. 1a,b), and therefore we also estimated $r(t)$ in the reverse direction (blue line and regions in Supplementary Fig. 1a,b). For the estimate of the initial r_0, we averaged the estimates from the forward and reverse time series. For the scenario of step changes in $R(t)$ (Supplementary Fig. 1c), the estimates were unbiased and had accurate confidence intervals, although for the scenario of gradual changes (Supplementary Fig. 1d), there was some downwards bias. Nonetheless, the estimates of initial R_0 captured the order of simulations according to the true R_0. In contrast, fitting the same time series with a commonly used Bayesian model that incorporates the transmission process given in the R package EpiEstim[7] gave estimates that poorly reflect the true (simulated) initial R_0 (Supplementary Fig. 1e,f).

We also used the simulation model to investigate the properties of the statistical method when the number of deaths was low, as occurred in some time series. Reducing the simulated values of R_0 reveals that the estimates of r_0 become biased downwards when the maximum number of reported deaths per day drops below 15 (Supplementary Fig. 2a). This is due to the time series containing too little information about the rate of increase in the number of mortalities for accurate estimates. Because we did not think that our method (or any other) could overcome this challenge, we incorporated population size encompassed by a time series in the

subsequent regression analysis. We used population size rather than the maximum number of deaths, because this would introduce a confounding effect: time series with higher r_0 will likely have higher numbers of deaths.

In order to extrapolate the estimates of R_0 from 160 time series to the remaining counties in the conterminous USA, we *a priori* selected four predictors. We selected population size encompassed by the time series to account for possible downwards bias in sparse datasets. We selected the Julian date of the outbreak onset to factor out public and private responses to COVID-19. We included population density, because it could potentially affect transmission rates. Population size and density were weakly and negatively correlated among the 160 time series (Pearson correlation between log population size and log density = –0.25), and therefore there were no problems with multicollinearity. Finally, the regression model included spatial autocorrelation based on the latitude and longitude of the population-weighted midpoint of the counties or county aggregates. Because the regression model had residual variance that was only slightly higher than the variance of the estimates of r_0 that the regression predicted, the precision of the estimates from the regression for the counties without time series will be on par with the precision of the counties with time series.

Simulation model

To assess the robustness of the statistical model, we built a simulation model of a hypothetical epidemic. The simulation model tracks the epidemic on a daily time scale and explicitly includes the time period from infection to subsequent transmission (infectiousness), and from infection to death; therefore, it is akin to a SEIR model. The simulation model was not the same as the statistical model, so the goal was to determine whether the phenomenological statistical model was capable of capturing the rate of infection spread in the process-based simulations.

The simulation model tracks the number of infected individuals on day t who were infected τ days previously, $X(t;\tau)$. After 25 days, they are all assumed to be recovered or dead. The probability distribution of the day on which a susceptible is infected, $p(t)$, is given by a Weibull distribution with mean 7.5 days and standard deviation 3.4[6] (Supplementary Fig. 3a). For an individual who dies, the day of death, $d(t)$, is given by a Weibull distribution with mean 18.5 days and standard deviation 3.4[6] (Supplementary Fig. 3b). Finally, for case data we need to

know the time between initial infection and diagnosis, $h(t)$, which we assume is lognormally distributed with mean 5.5 days and standard deviation 2.2[8] (Supplementary Fig. 3c).

On day t, the number of new infections produced by individuals who were infected τ days earlier is $b(t)\, p(\tau)$. The term $b(t)$ is closely related to $R(t)$, the number of secondary infections caused per infection. However, because we allow $b(t)$ to fluctuate on a daily basis, here we use a notation that differs from $R(t)$. Note, however, that on average $R(t) = \sum_\tau b(t + \tau)\, p(\tau)$. The total number of new infections on day t is given by a lognormal Poisson distribution in which the mean of the Poisson process is $b(t)\, \alpha(t) \sum_\tau p(\tau)X(t;\tau)$, where the lognormal random variable $\alpha(t)$ is included to represent environmental variation.

Deaths occur according to a binomial distribution for each infection age category $X(t;\tau)$, so that the probability of death of individuals that had been infected τ days earlier is $(1 - s)\, \beta(t)$ $d(\tau)$, where s is the overall survival probability and $\beta(t)$ is a lognormal distribution. We assume that the overall survival probability for COVID-19 is 98%; changes in this assumption had little effect on the simulation study. Once an individual dies, they are removed from the pool of individuals.

To illustrate the simulations, we assumed that the expectation of the infection rate, $b(t)$, changes as a step function (Supplementary Fig. 4a, black line), while there is also daily variation around this expectation (Supplementary Fig. 4a, points). We also calculated $R(t)$ from the asymptotic rate of disease spread (Supplementary Fig. 4a, red line). This shows that the expected daily infection rate, $b(t)$, is closely related to the population-level $R(t)$. Over the simulated time series of 60 days, we then recorded the number of deaths (Supplementary Fig. 4b) and diagnosed cases (Supplementary Fig. 4c). We initiated the simulation with a single cohort of individuals, all infected on day 1 (Supplementary Fig. 4c, filled black dot). This gives the "worst-case" situation in which the distribution of time-since-infection is far from the stable age distribution.

We fit this simulated dataset using the same procedure as we used for the real data, including the same rules to determine which day to initiate the fitted time series (Supplementary Fig. 1a). We performed a similar exercise while assuming that the expectation of the infection rate, $b(t)$ changes geometrically, producing a linear change in $r(t)$ (Supplementary Fig. 1b). In this particular example, the estimated values of $r(t)$ are below the true values in the simulation in the first part of the time series. Because there was a lag in response of the estimates of $r(t)$

relative to $b(t)$, we fit the time series in both the forward and reversed directions, and we averaged these values (and their confidence intervals) for the final estimates. Note that this is possible in our approach, because we estimate $r(t)$ rather than $R(t)$.

We performed 100 simulations with the expectation of $b(t)$ changing as either a step function (Supplementary Fig. 1c) or geometrically (Supplementary Fig. 1d), to assess the overall robustness of the modeling approach. Simulations were performed by changing the initial value of $b(t)$. Because higher values of $b(t)$ led to much higher numbers of deaths, we shorted the intervals between step changes and increased the decline in geometric changes in $b(t)$ to roughly match the observed time series. Specifically, the simulated time series ranged in length from 55 to 150 days: for the case of step changes, the time series were broken into three equal periods, and for the case of geometric changes, the ending value of $b(t)$ was kept the same. We also estimated $R(t)$ using the R package EpiEstim under default control parameters[7]. EpiEstim has the same general structure of many of the Bayesian models that estimate $R(t)$ directly using information about the transmission process (Supplementary Fig. 1e,f). Even though EpiEstim is structurally more complicated than our model, it tended to give values of R_0 that were biased upwards when the true value was low, and biased downward when the true value was high. Finally, we investigated the bias in our estimates of r_0 when the maximum number of deaths in a time series was low by simulating time series for 20 to 70 days, using an initial value of $b(t)$ to correspond to $R_0 = 4$, and changing the timing of step changes or the rate of geometric decline of $b(t)$ to correspond to the length of the time series. The simulations show that the estimates of r_0 are downward biased when the total numbers of counts are low (Supplementary Fig. 2).

Analysis of Nextstrain metadata of SARS-CoV-2 strains

In the analyses presented in the main text, we used the GISAID metadata to test the specific assumption that the G614 mutation increases the rate of spread of SARS-CoV-2. Prior to this analysis, however, we analyzed a subset of the genomic data available from Nextstrain[9]. We present this analysis here, because it was a naïve analysis that did not have a specific hypothesis about what strains might lead to higher spread rates. Instead, we asked whether the proportion of different Nextstrain clades (19A, 19B, 20A, 20B, 20C in the USA) within a population were related to r_0 estimates. We used the same statistical approach as we present for the GISAID

metadata, except we included the proportion of strains from clades 19A, 19B, 20A, and 20B instead of the proportion in the G clades containing mutation G614; we excluded the largest clade, 20C, because the sums of the proportions must add to one, and therefore all of the information about the distribution of strain 20C among states is contained in the distribution of the other clades. We found that the proportion of samples within clade 19B had a negative effect on r_0 (P = 0.019, Supplementary Table 3). The high proportion of strains from 19B in the Pacific Northwest and the Southeast were associated with lower values of r_0 (Supplementary Fig. 5). Strain 19A, however, also does not contain the G614 mutation, and it did not have a negative effect on r_0.

Supplementary Figures and Tables

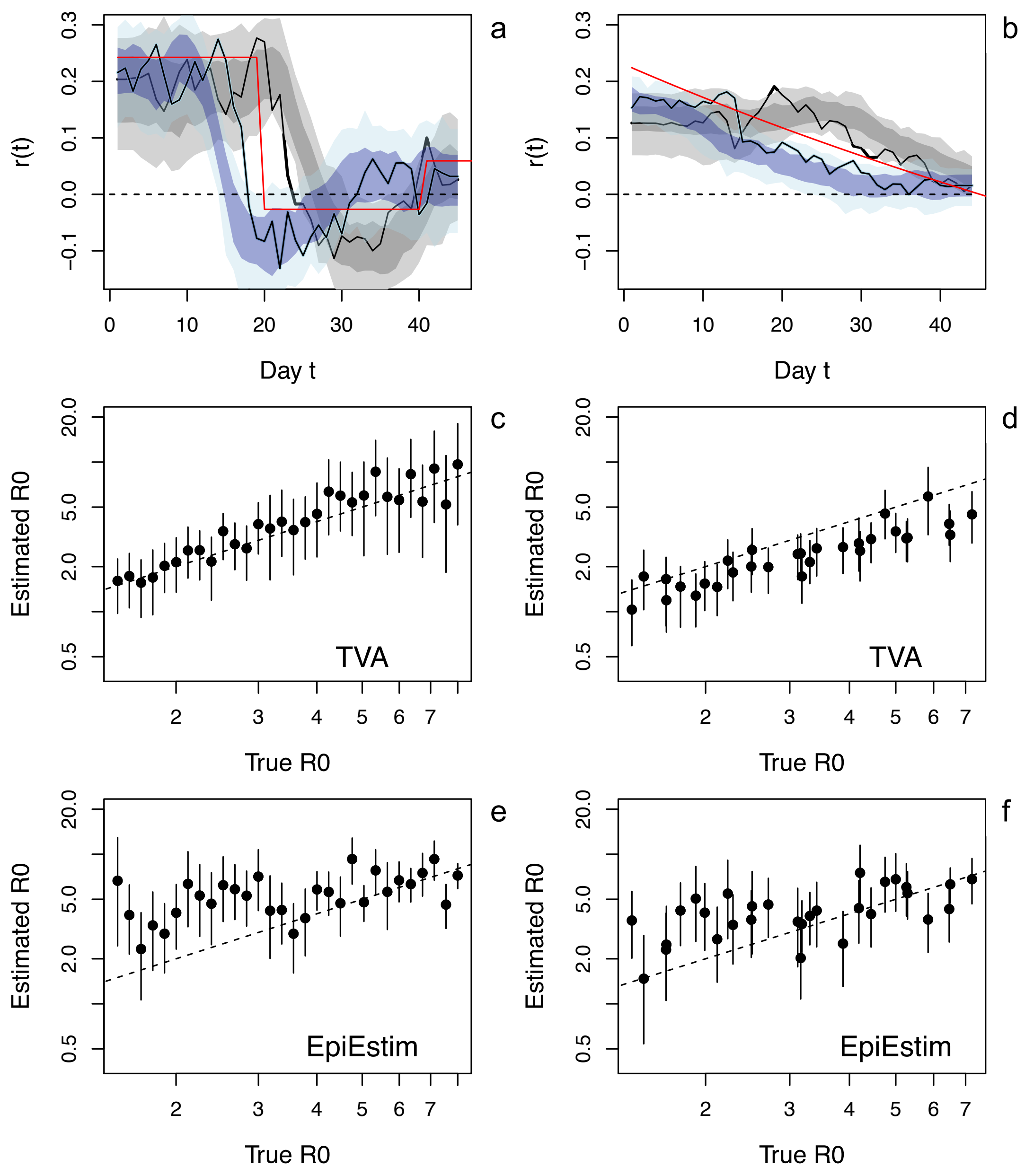

Supplementary Figure 1. Simulation study of fitting methods to epidemic death data. Simulations were fit with the time-varying autoregression model (TVA) in the forward (black line with dark and light gray regions giving 66% and 95% approximate confidence intervals) and reverse (blue line and regions – the light blue regions are sometimes obscured) directions when the true value of R(t) (red line) shows either **a** a step or **b** gradual changes. For each simulation, the forward and reverse estimates were averaged to give an estimate of R_0 with 95% confidence intervals, which are plotted against the true values of R_0 for step **c** and gradual **d** changes in R(t). **e,f** The same simulations with fit using EpiEstim.

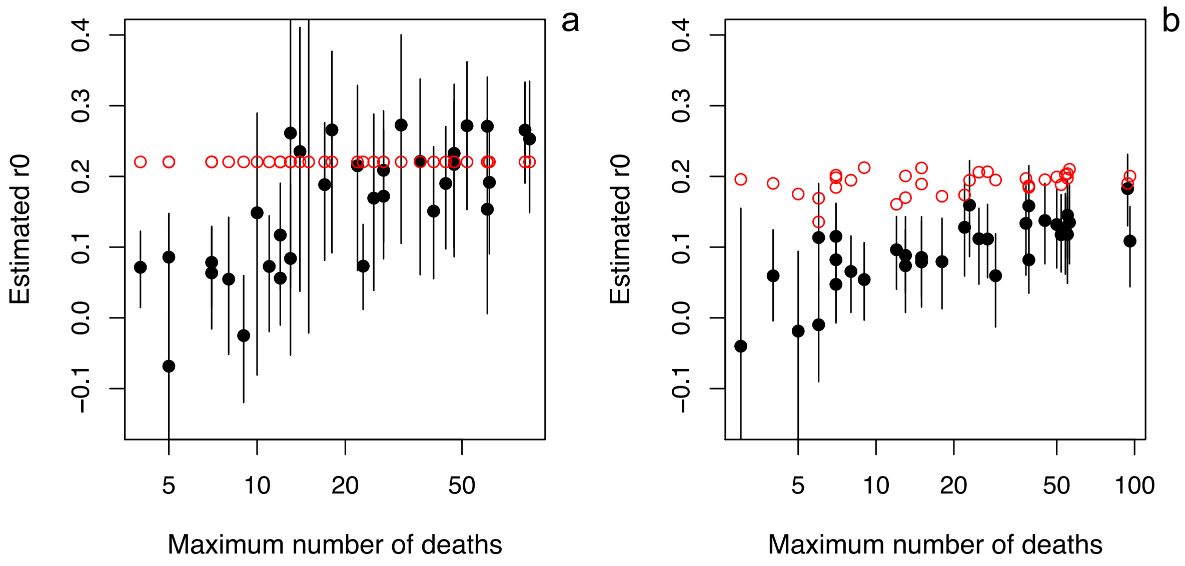

Supplementary Figure 2. Simulation study of the estimation of r_0 from the forward and reverse time-varying autoregressive model for different population sizes. Simulations following those used for Supplementary Figure 1 were performed assuming $r(t)$ changed either **a** in steps or **b** gradually. The simulations were performed using the same initial value of r_0, but the length of time of the simulation was varied to change the maximum number of deaths that occurred. Due to the stochastic nature of the simulations, the realized value of r_0 when the analysis was started differed among time series when $r(t)$ changed gradually (red points in **b**), while they were all 0.22 when $r(t)$ was changed in steps **a**. The median in the maximum number of deaths among the real county time series was 21.

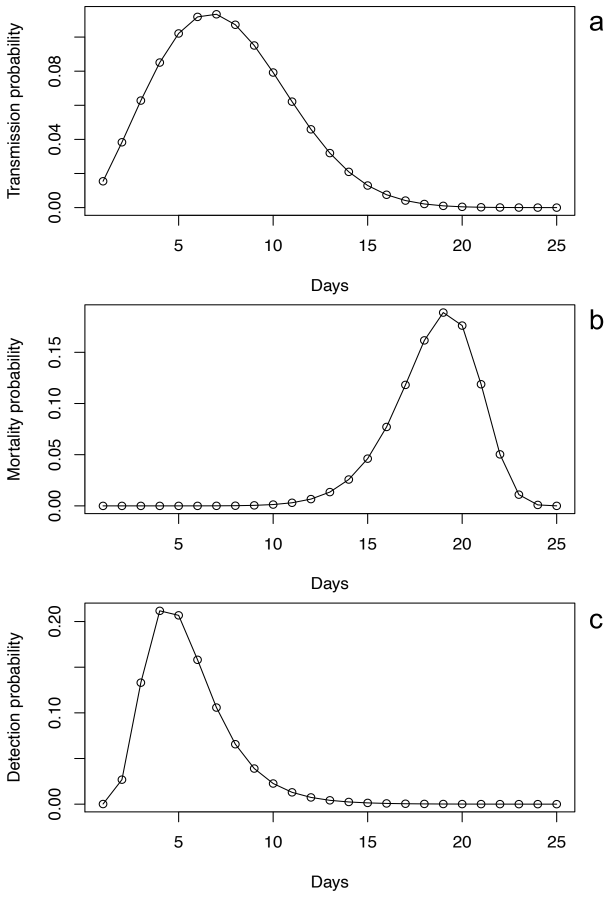

Supplementary Figure 3. Probability distributions used in the process-based simulation model used to test methods for robustness. **a** The probability distribution of the day on which a susceptible is infected, $p(t)$, given by a Weibull distribution with mean 7.5 days and standard deviation 3.4. **b** For an individual who dies, the day of death, $d(t)$, which is given by a Weibull distribution with mean 18.5 days and standard deviation 3.4. **c** For case data, the time between initial infection and diagnosis, $h(t)$, which is lognormally distributed with mean 5.5 days and standard deviation 2.2.

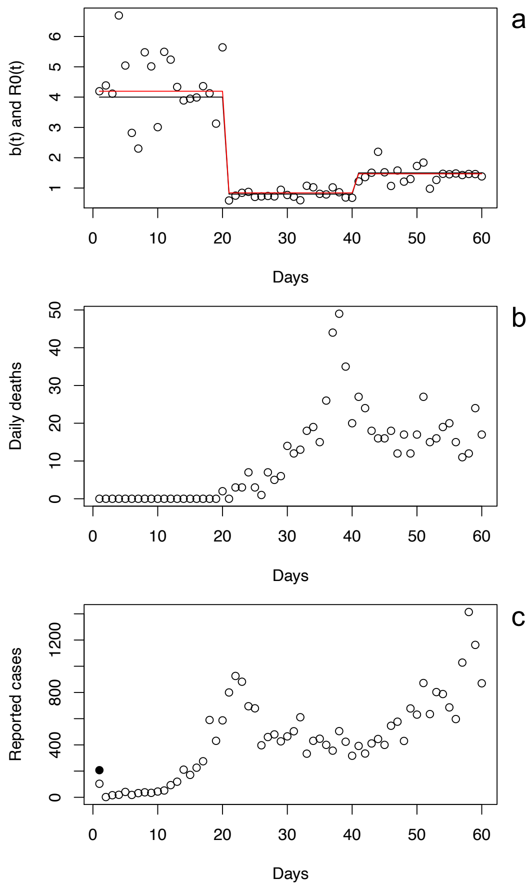

Supplementary Figure 4. Example simulation from the process-based model. **c)** Changes in the infection rate, $b(t)$, are modeled as a step function (black line) with daily variation (points). $R(t)$ (red line) tracks changes in $b(t)$. **b** and **c** The number of deaths (**b**) and diagnosed cases (**c**) when the simulation is initiated with a single cohort of individuals, all infected on day 1 (solid black dot).

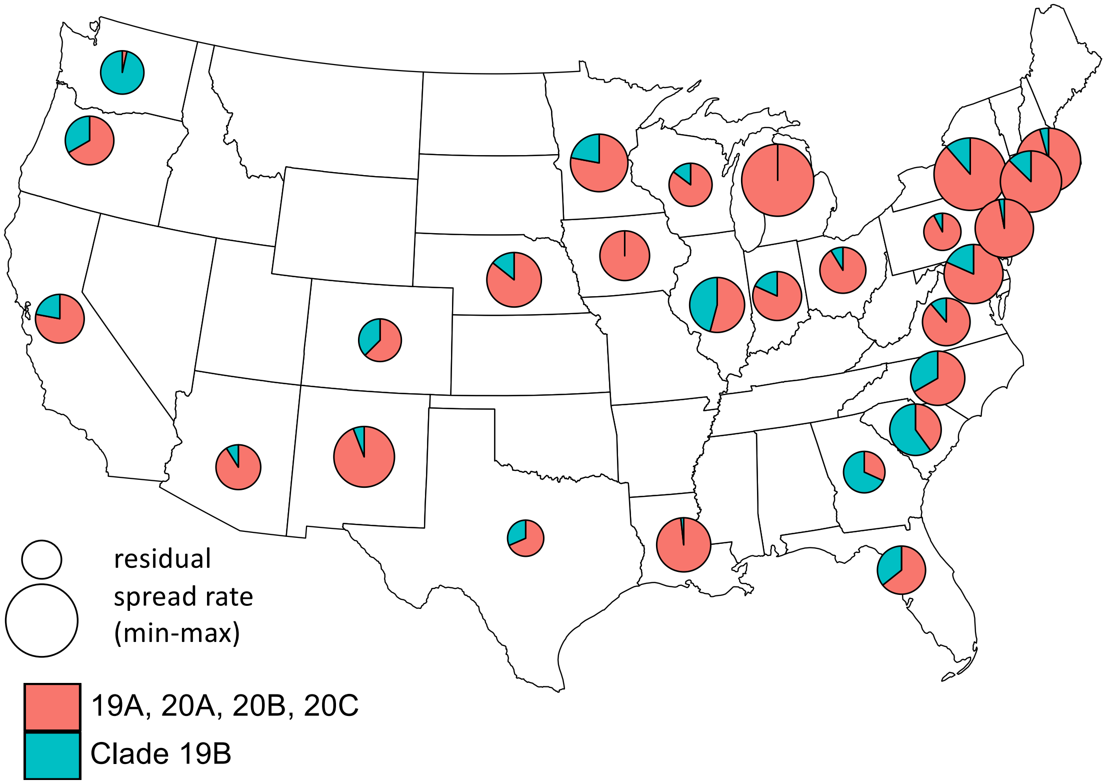

Supplementary Figure 5. Spatial distribution of the 19B clade of SARS-CoV-2 at the outbreak onset among states. Pie charts give the proportion of samples in states collected within 30 days following the outbreak onset that are in the 19B clade (blue). The size of the pie is proportional to the residual values of r_0 after removing the effects of the timing of outbreak onset, population size represented by the time series, and population density. For each state, we used the estimate of r_0 corresponding to the county or county-aggregate that had the greatest number of deaths.

Supplementary Data 1 (metadata). Spreadsheet giving COVID-19 spread rates and R0 at the onset of the epidemic for 3109 counties in the conterminous USA. Below are the included variables.

Variable	Description
ST	two-letter state abbreviation
state_county	state abbreviation with county name
fips	FIPS identifier for counties
lon	longitude
lat	latitude
den	population density
r0.est	estimate of r_0 from time-series analyses
r0.est.cor	corrected estimate of r_0 removing start.date and the population size
r0.l66.cor	lower 66% confidence interval of the corrected estimate of r_0
r0.u66.cor	upper 66% confidence interval of the corrected estimate of r_0
r0.pred	predicted estimate of r_0 from the regression model
r0.pred.se	standard error of the predicted estimate of r_0
R0.pred	predicted estimate of R_0 from the predicted estimate of r_0
R0.pred.l66	lower 66% confidence interval of the predicted estimate of R_0
R0.pred.u66	upper 66% confidence interval of the predicted estimate of R_0

Supplementary Table 1. Effect of the G514 mutation on the spread of COVID-19. Regression of the initial spread rate, r_0, of COVID-19 against (i) the date of outbreak onset, (ii) total population size, (iii) population density, and (iv) the proportion of samples of SARS-CoV-2 containing the G614 mutation in the spike gene[10]. The estimates of r_0 were for the county or county-aggregate with the greatest number of deaths in the state. All genetic samples were collected within 30 days following the onset of outbreak in a county. Twenty-eight states had five or more genetic samples, and only these states are included in the regression. Transforms of population size and density were selected to best-fit the data and satisfy linearity assumptions.

	**Coefficient**	**SE**	**t**	**P**
**onset**	–0.0032	0.0013	–2.42	0.024
**log(size)**	0.020	0.009	2.14	0.043
**density$^{1/4}$**	0.012	0.005	2.28	0.033
**G614**	0.133	0.051	2.61	0.016

Supplementary Table 2. Effect of Nextstrain SARS-CoV-2 clades on the spread of COVID-19. Regression of the initial spread rate, r_0, of COVID-19 against (i) the date of outbreak onset, (ii) total population size, (iii) population density, and (iv) the proportion of samples of SARS-CoV-2 in four of the five clades identified in the Nextstrain metadata[11]. The estimates of r_0 were for the county or county-aggregate with the greatest number of deaths in the state. All genetic samples were collected within 30 days following the onset of outbreak in a county. Twenty-seven states had five or more genetic samples, and only these states are included in the regression. Transforms of population size and density were selected to best-fit the data and satisfy linearity assumptions.

	**Coefficient**	**SE**	**t**	**P**
**onset**	–0.0032	0.0015	–2.2	0.040
**log(size)**	0.019	0.011	1.82	0.085
**density$^{1/4}$**	0.015	0.006	2.68	0.015
**19A**	-0.050	0.095	-0.53	0.60
**19B**	-0.147	0.054	-02.72	0.014
**20A**	-0.031	0.057	-0.54	0.59
**20B**	0.009	0.173	0.05	0.96

Supplementary Table 3. Variables giving population characteristics for counties in the conterminous USA. These variables were included in the regression model to assess the importance of population density and spatial autocorrelation in the estimation of r_0[12-19].

Variable	Description
age	proportion of the population over 65 years old, 2011-2015
adult obesity	incidence of adult obesity, 2015
diabetes	incidence of adult diabetes, 2015
education	percent bachelor's degree or higher, 2011-2015
income	median earnings 2011-2015
poverty	percentage people below federal poverty threshold, 2011-2015
economic equality	Gini index, 2013-14
race	percent White, non-Latino, 2015
political leaning	proportion of votes cast for Donald Trump, 2016

Supplementary Table 4. Factors explaining the post-intervention spread rate of COVID-19 for 160 county and county-aggregates in the USA. For 160 county and county-aggregates, regression of spread rate at the end of the time series, corresponding to 5 May, 2020, $r(t_{end})$, against (i) the date of outbreak onset, (ii) total population size and (iii) population density, in which (iv) spatial autocorrelation is incorporated into the residual error. Transforms of population size and density were selected to best-fit the data and satisfy linearity assumptions. The coefficient column contains the estimate of the regression parameters with their associated t-tests; spatial autocorrelation is characterized by a range and nugget for regional and local sources of variation, and their joint significance is given by a likelihood ratio test. For the overall model, $R^2_{pred} = 0.40$.

	**Coefficient**	**SE**	**t**	**P**	**partial R^2_{pred}**
**onset**	0.0020	0.0003	6.18	$< 10^{-8}$	0.15
**log(size)**	0.0093	0.0021	4.43	$< 10^{-6}$	0.070
**density$^{1/4}$**	-0.0010	0.0014	-0.68	0.50	0.003
**space**	range = 0.24<br>nugget = 0		$\chi^2_2 = 13.89$	0.001	0.13

Supplementary References

1 Flaxman, S. *et al.* Estimating the effects of non-pharmaceutical interventions on COVID-19 in Europe. *Nature* **584**, 257-261 (2020).

2 Scire, J. *et al.* Reproductive number of the COVID-19 epidemic in Switzerland with a focus on the Cantons of Basel-Stadt and Basel-Landschaft. *Swiss Medical Weekly* **150**, w20271 smw.ch/article/doi/smw.2020.20271 (2020).

3 Gelman, A. & Hill, J. *Data analysis using regression and multilevel/hierarchical models*. (Cambridge University Press, 2007).

4 Efron, B. & Tibshirani, R. J. *An introduction to the bootstrap*. (Chapman and Hall, 1993).

5 Dublin, L. I. & Lotka, A. J. On the true rate of natural increase. *Journal of the American Statistical Association* **20**, 305–339 (1925).

6 Li, Q. *et al.* Early transmission dynamics in Wuhan, China, of novel coronavirus–infected pneumonia. *New England Journal of Medicine* **382**, 1199-1207 (2020).

7 Cori, A., Ferguson, N. M., Fraser, C. & Cauchemez, S. A new framework and software to estimate time-varying reproduction numbers during epidemics. *American Journal of Epidemiology* **178**, 1505-1512 (2013).

8 Ferretti, L. *et al.* Quantifying SARS-CoV-2 transmission suggests epidemic control with digital contact tracing. *Science* **368**, eabb6936 (2020).

9 Hadfield, J. *et al.* Nextstrain: real-time tracking of pathogen evolution. *Bioinformatics* **34**, 4121-4123 (2018).

10 Elbe, S. & Buckland-Merrett, G. Data, disease and diplomacy: GISAID's innovative contribution to global health. *Global Challenges* **1:33-46** (2017).

11 NextstrainTeam. Nextstrain. https://nextstrain.org/ncov (2020).

12 Measure of America. Mapping America: Safety & security indicators. http://measureofamerica.org (2018).

13 Measure of America. Mapping America: Education indicators. http://measureofamerica.org (2018).

14 Measure of America. Mapping America: Demographic indicators. http://measureofamerica.org (2018).

15 Measure of America. Mapping America: Health indicators. http://measureofamerica.org (2018).

16 Measure of America. Mapping America: Work, wealth & poverty indicators. http://measureofamerica.org (2018).

17 MIT Election Data and Science Lab. County Presidential Election Returns 2000-2016. 10.7910/DVN/VOQCHQ (2018).

18 Skinner, B. T. Making the connection: Broadband access and online course enrollment at public open admissions institutions. *Research in Higher Education* **60**, 960-999 (2019).

19 Measure of America. HD Index and supplemental indicators by county, 2013-2014 dataset. http://measureofamerica.org (2013).
