# Supplementary material for: Estimating and explaining the spread of COVID-19 at the county level in the USA
Source: Commun Biol. 2021 Jan 5;4:60. doi: 10.1038/s42003-020-01609-6 (PMC7785728; doi:10.1038/s42003-020-01609-6)
Supplement: Supplementary file 3 — Description of Supplementary Files [file 42003_2020_1609_MOESM3_ESM.pdf]

Description of Additional Supplementary Files

**File name:** Supplementary Data 1

**Description:** Spreadsheet giving COVID-19 spread rates and R0 at the onset of the epidemic for 3109 counties in the conterminous USA.
